# *Schistosoma haematobium* and *Schistosoma bovis* first generation hybrids undergo gene expressions changes consistent with species compatibility and heterosis

**DOI:** 10.1371/journal.pntd.0012267

**Published:** 2024-07-02

**Authors:** Eglantine Mathieu-Bégné, Julien Kincaid-Smith, Cristian Chaparro, Jean-François Allienne, Olivier Rey, Jérôme Boissier, Eve Toulza

**Affiliations:** 1 Department of Environmental Sciences, Zoology, University of Basel, Basel, Switzerland; 2 IHPE, Université de Montpellier, CNRS, Ifremer, Université Perpignan Via Domitia, Perpignan, France; 3 CBGP, IRD, CIRAD, INRAE, Institut Agro, Université de Montpellier, Montpellier, France; University of Glasgow, UNITED KINGDOM

## Abstract

When two species hybridize, the two parental genomes are brought together and some alleles might interact for the first time. To date, the extent of the transcriptomic changes in first hybrid generations, along with their functional outcome constitute an important knowledge gap, especially in parasite species. Here we explored the molecular and functional outcomes of hybridization in first-generation hybrids between the blood fluke parasites *Schistosoma haematobium* and *S*. *bovis*. Through a transcriptomic approach, we measured gene expression in both parental species and hybrids. We described and quantified expression profiles encountered in hybrids along with the main biological processes impacted. Up to 7,100 genes fell into a particular hybrid expression profile (intermediate between the parental expression levels, over-expressed, under-expressed, or expressed like one of the parental lines). Most of these genes were different depending on the direction of the parental cross (*S*. *bovis* mother and *S*. *haematobium* father or the reverse) and depending on the sex. For a given sex and cross direction, the vast majority of genes were hence unassigned to a hybrid expression profile: either they were differentially expressed genes but not typical of any hybrid expression profiles or they were not differentially expressed neither between hybrids and parental lines nor between parental lines. The most prevalent profile of gene expression in hybrids was the intermediate one (24% of investigated genes). These results suggest that transcriptomic compatibility between *S*. *haematobium* and *S*. *bovis* remains quite high. We also found support for an over-dominance model (over- and under-expressed genes in hybrids compared to parental lines) potentially associated with heterosis. In females in particular, processes such as reproductive processes, metabolism and cell interactions as well as signaling pathways were indeed affected. Our study hence provides new insight on the biology of *Schistosoma* hybrids with evidences supporting compatibility and heterosis.

## Author summary

When two species, composed of different parental genetic material, manage to produce a viable offspring, new allelic interactions might arise. We do not know much about how genes are expressed in such hybrids compared to their parental species, especially for parasite species. Here we investigated gene expressions in first generation hybrids of two blood fluke parasites: *Schistosoma haematobium* and *S*. *bovis*. We quantified and assigned in five typical profiles genes expressed in first generation hybrids (i.e., intermediate between the parental expression levels, over-expressed, under-expressed, or expressed like one of the parental lines) and investigated their associated biological processes. Gene expressions in hybrids revealed a substantial fraction of genes being unassigned (once the sex and the direction of the cross from which a hybrid resulted were controlled) which were differentially expressed genes but not typical of any expression profiles or were not differentially expressed neither between hybrids and parental lines nor between parental lines. Among assigned genes, we recorded a predominance of genes being expressed at an intermediate level in hybrids compared to both parental species, hence highlighting the compatibility between the two species. Interestingly, we found, in particular in female hybrids, genes over- and under-expressed compared to parental lines. Those synergetic expression profiles are usually associated with heterosis in a model called over-dominance. At the functional level, our results provide evidence for compatibility between parental lines and potential heterosis in hybrids with biological processes expressed in hybrids involved in signaling pathways, energy intake, and reproduction.

## Introduction

Species definition has for long fueled debates among biologists due to the difficulty to draw clear boundaries between such biological entities [1]. The study of hybridization offers the possibility to shed light on the process of species formation. So far, hybridization has been mostly studied in terms of populations structure, genome structure and phenotypic outcomes [2,3]. However, the molecular mechanisms underlying the viability and performance of hybrids still constitute an important knowledge gap, especially regarding empirical evidence. In particular, there are significant research opportunities in studying the gene expression, epistasis and epigenetics marks of hybrids that arise from non-model parental lines (i.e., ecologically realistic hybrids) that have to date been less studied than model laboratory species or species of economic interest [4–6].

The study of hybridization has been mostly motivated by two extreme phenotypes in hybrids, namely hybrid incompatibilities and heterosis (or hybrid vigor, [7]). Hybrid incompatibilities describes the case when the hybrid phenotype cannot be maintained or achieved due to conflicts in the expression of each parental species genomes (i.e., *genome clash*, [7]). At the other end of the spectrum, heterosis is a phenotype characterized by higher performance of the hybrids compared to parental lines in term of growth, size or reproductive potential [8,9]. Heterosis may provide a particular benefit in farmed species because it increases their yield, explaining why a substantial part of the literature on heterosis has focused on species of agroeconomic interest. Most first-generation hybrids are sterile or non-viable at all due to parental genome conflicts, though when viable they usually display vigor on at least some traits [7]. Interestingly, if heterosis can emerge in the first generation of hybridization, this phenotype is not maintained through subsequent generations that usually show a decrease in heterozygosity (which get them closer to the parental phenotype) and may ultimately display hybrid breakdown (i.e., weaker performance than both parental lines, [10]). Hence the “hybrid phenotype” is a dynamic process throughout hybrid generations that depends on the interaction of alleles that are brought together at each generation [8].

Similarly, the process of hybridization does not occur equally among species since permeability toward hybridization may drastically vary between genomic backgrounds. Even after millions of years some species still display significant levels of admixture whereas other species, although close genetically, have evolved strong reproductive isolation mechanisms that prohibit hybridization either at early stages of reproduction (pre-zygotic barriers) or along the hybrid lifespan (post-zygotic barriers, for instance when hybrids die prematurely or are sterile) [11]. Once successful fertilization has occurred, producing a viable hybrid zygote depends on parental genomes’ interaction and environmental selection. For instance, differences in the karyotype generally results in non-compatible genome interaction and non-viable offspring [12]. At the gene level, when two species hybridize, some alleles that have not been brought together might interact to result in an hybrid phenotype [13]. Depending on the degree of genetic divergence between parental lines (and how conserved their transcription factors are), hybrids might form and prosper or might just not be viable [13]. Some theoretical gene-level models have been developed to explain exacerbated hybrid phenotypes such as heterosis (eg., dominance and over-dominance models which expect either selective expression of the most advantageous parental allele or synergetic expression of parental alleles respectively [9]). Interestingly, incompatibility is also expected to be associated with more over- and under-expressed genes in hybrid than intermediate level of expression compared to parental lines, [13]. However, those theoretical expectations remain mostly explored in species of agronomic interests and provided sometime contradictory results [13]. For species hybridizing naturally with significant introgression levels, even description of gene expression patterns remains to date rarely documented.

Among naturally hybridizing species, there is a growing interest in parasites and/or their vectors since their geographic range can be affected by global changes (including human migration, habitat loss, global trade and climate change), hence bringing together pathogens that were previously not in contact [14]. This is particularly worrying due to the potential for exacerbated virulence in hybrid parasites [15]. Viable hybridization in parasites often results in stronger detrimental effects on their hosts, but also broader vector/host range, which carry the potential for zoonotic transmission when parasites of wild or farmed animals interact with human parasites [14]. For instance, introgression between two subspecies of *Trypanosoma brucei* have been shown to be associated with different levels of virulence [16]. Similarly, the high competence of malaria vectors, as revealed by their broad definitive host spectrum that encompassed various mammals and birds species, is thought to result from genetic admixture between *Anopheles* species which is inferred from their high levels of introgression [14,17,18]. To date most studies have focused on identifying and quantifying parasite hybrids in the wild. Although necessary to assess the risks associated with parasite hybridization, it is currently very rare for studies to focus on the molecular bases of the hybrid phenotypes in parasites (but see [19]).

*Schistosoma haematobium* and *S*. *bovis* are two blood fluke parasites for which hybrid vigor has been documented in the laboratory [20] and is suspected to have impacts on human morbidity in the field [21]. Schistosomes are the etiologic agents of Schistosomiasis (or Bilharziasis) a Neglected Tropical Disease affecting over 240 million people in the world [22]. These gonochoric parasites have a two-host life cycle including a mollusc intermediate host in which asexual multiplication occurs, and a mammalian definitive host in which sexual reproduction (separate sexes) takes place. In the latter host, parasite species co-infection can lead to interbreeding and the production of viable hybrid progeny [23,24]. Actually, some recent studies have revealed various levels of introgression notably in *S*. *haematobium* from different other species of *Schistosoma* including *S*. *bovis* and most likely following a scenario of ancient introgression [25–27]. However, the question of potential ongoing hybridization is still open and more knowledge is required on those potential hybrids to better understand their impact on future transmission dynamics of schistosomiasis. Several field or experimental viable interspecific crosses have indeed been evidenced among the *Schistosoma* genus [28]. Depending on the phylogenetic distance between the interacting species, the genetic background of the resulting progeny varies between parthenogenic (hence non-viable introgressed individuals) to fully viable introgressed individuals [29]. *Schistosoma haematobium* and *S*. *bovis* are sister species amongst the *haematobium* clade [30]. *Schistosoma haematobium* is specialized toward humans as definitive hosts, while *S*. *bovis* is a livestock and rodent parasite. They also display differences in their tropism since *S*. *haematobium* is urogenital while *S*. *bovis* is located in the mesenteric vein system. However, despite apparent host-specific barriers, hybrids between *S*. *haematobium* and *S*. *bovis* are observed in the field with common occurrences in several African countries including Senegal, Benin, Mali, Niger, Cameroon, Ivory Coast or Nigeria [27,31–37]. *Schistosoma haematobium x S*. *bovis* hybrids were also involved in the European outbreak that occurred in Corsica in 2013 and where infections are still persisting and expanding to nearby rivers [38,39]. Both for human health impacts and epidemiological aspects, schistosome hybrids raise worrying issues. Schistosome first-generations hybrids tend to produce both greater number of eggs and sometimes larger or longer eggs than parental forms [27,28,40,41] and the presence of eggs trapped in human tissues are responsible for the physiopathology of the disease. Hybridization among schistosomes is hence expected to affect parasite-induced pathology and transmission with important consequences for public health and disease control. Past studies on schistosome hybrids have focused on describing their distribution and occurrence in natural populations, here, we attempt to provide new insight on some of the molecular changes occurring following first-generation hybridization between *S*. *haematobium* and *S*. *bovis*.

In this study we aimed at characterizing gene expression levels and molecular pathways that vary in *S*. *haematobium* and *S*. *bovis* first generation hybrids compared to parental species. Relying on a genome-guided transcriptomic approach we first assembled transcripts from *S*. *haematobium*, *S*. *bovis* and their hybrids and compared their gene expression levels using *S*. *haematobium* genome as reference. We categorized hybrid expression profiles by comparing them to both parental lines. Hence, we distinguished five expression profiles of the hybrids namely: over-expressed, under-expressed, intermediate (i.e., intermediate between the parental expression levels also referred as additive expression, [42,43]), *S*. *haematobium-*like and *S*. *bovis-*like expression profiles. Finally, among genes belonging to each category of expression profiles we tested whether some biological processes were significantly enriched (i.e. over-represented). We expect to find evidence for conserved compatibility between *S*. *haematobium* and *S*. *bovis* due to their introgression history that could translate into a high portion of genes with intermediate levels of expression between parental lines (i.e., no synergetic or dominance effects). However, based on current evidence on heterosis in cultivated species and model organisms, we could also expect to observe some genes having a non-intermediate profile (over- or under-expressed and parent-like, known as over-dominance and dominance model of gene expression respectively) that may sustain an increased expression of hybrid vigour traits (such as reproduction or growth) when comparing the hybrids to at least one of the parental species. Note that here we refer to heterosis as the enhancement of some traits resulting of the mixing of genetic contribution from each parent in their offspring, whether the parental lines consist of two different species or two entities distinct enough to induce a substantial outbreeding event in their first-generation offspring. We hence consider that heterosis can apply for trait enhancement in *S*. *haematobium x S*. *bovis* offspring even in presence of introgression between parental lines as long as the two are distant enough and the introgression events ancient.

## Material and methods

### Ethics statement

Housing, feeding and animal care, including experiments on animals were carried out according to the national ethical standards established in the writ of 1 February 2013 (NOR: AGRG1238753A) setting the conditions for approval, planning and operation of establishments, breeders and suppliers of animals used for scientific purposes and controls. The experiments carried out for this study were approved and provided a permit A66040 for animal experimentation by the French Ministry of Agriculture and Fishery (Ministere de l’Agriculture et de la Peche), and the French Ministry for Higher Education, Research and Technology (Ministere de l’Education Nationale de la Recherché et de la Technologie). The investigator has the official certificate for animal experimentation, obtained from both ministries (Decret n° 87/848 du 19 octobre 1987; authorization number 007083). Parasite collection campaign is described in [37]. Specifically, the campaign was authorized by the National Ethics Committee of Cameroon (No. 2019/01/UDM/PR/CIE), the Ministry of Public Health and the Ministry of Basic Education and within each surveyed schools, by local administrative authorities, school inspectors, directors and teachers [37]. All patients found to be positive were treated in accordance with WHO recommendations.

### Study model

#### Parasite parental species and reciprocal experimental crosses

*Schistosoma haematobium* was initially recovered from infected patients in the Southeast part of Cameroon (Barombi Kotto lake, 4°28’04”N. 9°15’02”W) in 2015 [37]. Urine was collected during a Bilharziasis screening campaign and oral consent was obtained for each patient [37]. Eggs from positive urine samples were collected and we exposed local intermediate hosts (*Bulinus truncatus*) to five miracidia before they were transferred to the laboratory. Parasites were further maintained through completion of their cycle using Golden hamsters (*Mesocricetus auratus*) as definitive hosts and *B*. *truncatus* as intermediate host [24]. Because *S*. *haematobium* has recently been shown to be introgressed with *S*. *bovis* at various degrees [25,26] we took several precautions to ensure that the *S*. *haematobium* strain we used was as pure as possible (i.e., if introgression occurred it was far enough in the past to consider that the offspring between *S*. *haematobium* and *S*. *bovis* would undergo a significant outbreeding event at the first generation). First, this strain of *S*. *haematobium* was specifically collected in Barombi Kotto because of the absence of livestock hence limiting interspecific interactions with other *Schistosoma* species (in particular with *S*. *bovis*, [44,45]). To further test for the purity of *S*. *haematobium*, 3 cercariae from 13 infected molluscs were genotyped using 11 microsatellite markers and were compared to *S*. *haematobium* miracidia from human [37] and adult *S*. *bovis* collected from cattle [45] in Cameroun. All *S*. *haematobium* individuals clustered apart from the *S*. *bovis* samples with no indication of potential hybrids (S1 Fig). We further sequenced the COI gene from 35 schistosomes of this Barombi *S*. *haematobium* strain and all those sequences were referenced as *S*. *haematobium* sequences only (no sequence similar to other schistosome species were found, COI sequences are available in GenBank under accession numbers PP537975-PP538009). On the other hand, *S*. *bovis* was recovered from the Spanish laboratory of parasitology of the Institute of Natural Resources and Agrobiology in Salamanca, and maintained in the laboratory with both *Bulinus truncatus* and *Planorbarius metidjensis* sympatric intermediate hosts and on *M*. *auratus* definitive hosts [46].

Starting with experimental crosses, we first created a full-sibling inbred line of *S*. *haematobium* because of its potential high genetic diversity compared to the *S*. *bovis* isolate maintained in laboratory for several decades (i.e., inbred isolate maintained in the lab since the 71’s). A high genetic diversity in one of the parental lines would indeed impede our ability to detect differentially expressed genes due to a higher variance between samples. We hence crossed a single male with a single female after molecular sexing of the parasite larvae [47]. Briefly, batches of *B*. *truncatus* were individually infected with a single miracidium of *S*. *haematobium*, allowing the development of clonal male or female genotype. At patency (i.e., 55 days after exposure) cercariae genotypes emitted from individual molluscs were sexed and parasites from two molluscs (one infected with a male genotype and one with a female genotype) were used to infect hamsters (300 cercariae of each sex) by surface application method for one hour. The resulting offspring constituted our full-sibling inbred line used for subsequent experiments. Methods employed for mollusc and rodent infections and parasite collection were described previously [48–50].

To recover parental species adult worms and assess their gene expression profiles, we infected six hamsters with homospecific combinations of 600 cercariae (300 cercariae of each sex) of *S*. *haematobium*, or *S*. *bovis*. Briefly, for both parental species, a pool of *miracidia* obtained from homospecific crosses was used to infect molluscs (5 *miracidia* per mollusc). Fifty-five days after exposing the molluscs to the parasites, pools of 600 *cercariae* from infected molluscs were used to infect hamsters. Three months after infection hamsters were euthanatized and adult worms were recovered by hepatic perfusion.

**Fig 1:**
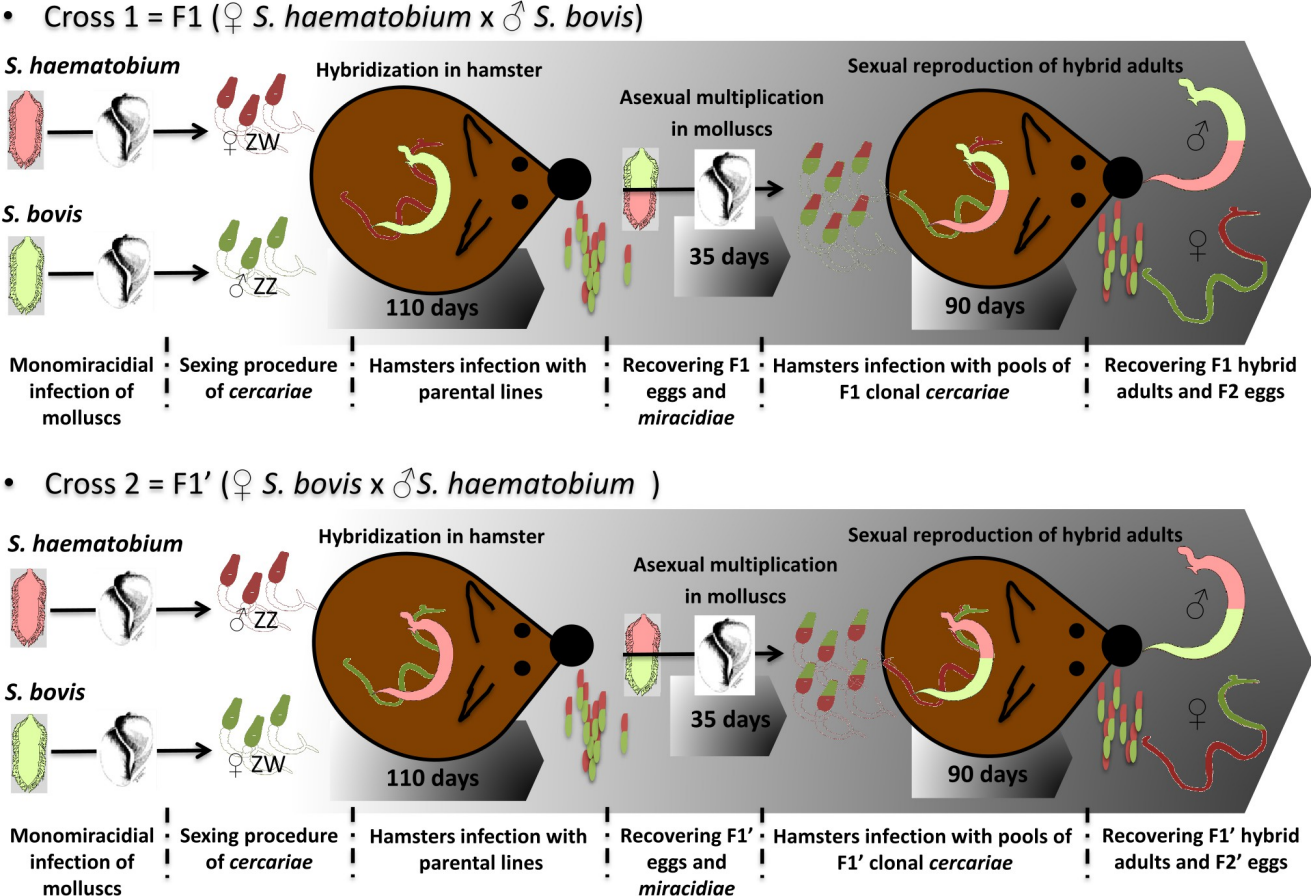
Experimental infections to produce *S*. *haematobium x S*. *bovis* reciprocal first-generation hybrids. Mollusc drawing credit: https://commons.wikimedia.org/w/index.php?curid=15147834, unchanged.

Laboratory *S*. *haematobium x S*. *bovis* hybrids were produced by exposing molluscs (*B*. *truncatus* or *P*. *metidjensis*, respectively) to a single miracidium of either parasite species and using the resulting single sex clonal pools of *cercariae* of each species to infect hamsters with mixed combinations of parasites in equal proportions (Fig 1). *Schistosoma haematobium* and *S*. *bovis* were reciprocally crossed to produce F1 (female *S*. *haematobium* x male *S*. *bovis*) and F1’(female *S*. *bovis* x male *S*. *haematobium*) hybrids. Batches of six hamsters per combinations were infected using 300 female *S*. *haematobium* and 300 male *S*. *bovis cercariae* (F1 cross; n = 6) or 300 male *S*. *haematobium* and 300 female *S*. *bovis cercariae* (F1’ cross; n = 6). Three months and twenty days after infection, hamsters containing the adult parents (heterospecific couples) and hybrid egg progenies were euthanized.

In order to recover first generation reciprocal F1 and F1’ hybrid adult worms and evaluate their expression profiles, hybrid eggs were hatched and hybrid *miracidia* were used to infect *B*. *truncatus* snails (n = 5 *miracidia* per mollusc). After the prepatent phase (~35 days) the F1 and F1’ *cercariae* were used to infect hamsters (n = 7 hamsters for each cross) with pools of 600 cercariae of unknown sex, and F1 and F1’ adult worms were collected from portal perfusion at 90 days after infection. A graphical representation of the experimental cross design is shown in Fig 1.

### RNA extraction and transcriptome sequencing procedure

Using a binocular microscope and a small paintbrush we separated the paired adult worms according to their sex to avoid any contamination from the other sex. We constituted pools of 10–12 individuals per condition (*S*. *haematobium*, *S*. *bovis*, F1 and F1’) and sex in three biological replicates representing a total of 24 samples (4 conditions x 2 sexes x 3 replicates). These samples were directly flash frozen in liquid nitrogen, and stored at -80°C. Total RNA extractions were performed using the TRIzol Thermo Fisher Scientific protocol (ref: 15596018) slightly modified as each reagents volume was halved. Briefly, pools of frozen adult worms in 2ml microtubes were ground with two steel balls using Retsch MM400 cryobrush (2 pulses at 300Hz for 15s). After extraction following manufacturer’s protocol, total RNA was eluted in 44 μl of ultrapure water. DNase treatment was then performed using Thermofisher Scientific Turbo DNA-free kit (ref: AM1907). RNA was then purified using the Qiagen RNeasy mini kit (ref: 74104) using manufacturer’s protocol and eluted in 42 μl of ultrapure water. Quality and concentration were assessed by spectrophotometry with the Agilent 2100 Bioanalyzer system and using the Agilent RNA 6000 nano kit (ref 5067–1511). Library construction and sequencing were performed at the Génome Quebec platform. The TruSeq stranded mRNA library construction kit (Illumina Inc. USA) was used following the manufacturer’s protocol with 300 ng of total RNA per sample. Sequencing of the 24 samples was performed in 2x100 bp paired-end on an Illumina HiSeq 4000. Sequencing data are available at the NCBI-SRA under the BioProject accession number PRJNA491632.

### Transcriptome assembly

We conducted a reference-based transcriptome assembly using *S*. *haematobium* reference genome [51] and the RNA-sequencing data generated in this study. Note that although *S*. *haematobium* was used as a reference, recent genomic characterization of this strain suggests that in fact it contains introgressed *S*. *bovis* alleles in genomic regions spanning up to 100 kb [52,53]. First reads were quality trimmed and adapter were removed using Trimmomatic (version 0.39 [54]) with default paired-end settings [54]. Reads were then mapped on the genome of *S*. *haematobium* using the splice aware mapper Hisat2 (version 10.3.0 [55]). Sam output were converted into bam, sorted and indexed using Samtools (version 1.16.1 [56]). In order to gather already referenced transcripts but also potentially new ones the software Stringtie (version 2.1.7 [57]) was used to assemble transcripts per sample while recovering intron/exon structure. Finally, Stringtie’s output files from each sample were merged in ordered to obtain congruent transcripts IDs across individual assemblies using the option—merge from Stringtie. This last step resulted in a transcriptome assembly of 66,012 transcripts and 16,840 genes while the reference genome of *S*. *haematobium* contains 14,698 transcripts and 9,431 protein-coding genes. Our assembly was compared to the reference gene models of *S*. *haematobium* to ensure its quality using Gffcompare (version 0.11.2 [58], S1 and S2 Tables).

All bioinformatics calculations were performed at sciCORE (http://scicore.unibas.ch/) scientific computing center at University of Basel.

### Quantification of gene expression and differential expression analysis

Gene expressions were quantified using the software FeatureCounts (version 2.0.3, from the Subread suite [59]) with mapped reads of each sample and the transcriptome assembly achieved in this study as input. A matrix of gene raw counts was outputted and used for further differential expression analysis conducted in R (version 4.0.0 [60]) and using the package DESeq2 (version 1.28.1 [61]). A brief inspection of our gene expression dataset was performed using a Principal Component Analysis and revealed that one F1 replicate suspiciously clustered apart from others and with the wrong sex cluster. We thus excluded this F1 replicate from further analysis (S2 Fig). We further filtered out genes with less than 10 counts across all samples hence resulting in a set of 15,770 analyzed genes over 35 samples (see results).

The R package DESeq2 that internally corrects the library size and allows calculating differential gene expression was used to determine gene expression profiles. Based on expression profiles we defined five categories of hybrid genes that were captured setting proper contrasts in DESeq2. The *under-expressed profile* refers to genes that were under-expressed in hybrids compared to both *S*. *haematobium* and *S*. *bovis* with a significance level defined at a False Discovery Rate of 5% (FDR<0.05). The *over-expressed profile* describes genes that are significantly over-expressed in hybrids compared to both *S*. *haematobium* and *S*. *bovis* (FDR<0.05). The *intermediate profile* encompasses genes that have an expression level between those of the parents, (i.e. genes that are significantly over-expressed in hybrids compared to one of the parental species while being significantly under-expressed compared to the other at a FDR<0.05). The *haematobium-like profile* describes genes that are significantly differentially expressed between *S*. *haematobium* and *S*. *bovis* (FDR<0.05 and Log2Fold Change (LFC)>1) but that are similarly expressed between hybrids and *S*. *haematobium* (Log2Fold Change (LFC)<0.05 and FDR>80%). Finally, the *bovis-like profile* refers to genes that are significantly differentially expressed between *S*. *haematobium* and *S*. *bovis* but that are similarly expressed between hybrids and *S*. *bovis*. The remaining unassigned genes included some for which the levels of expression did not differ significantly neither between parental lines nor between any parental line and hybrid offspring and/or genes with a significant differential expression but that did not fulfill the set of criteria (FDR thresholds and/or LFC) to be classified amongst the different hybrid expression profiles. To capture cross-specific and sex-specific gene expression patterns, hybrids expression profiles were quantified within each sex and each cross (i.e., F1 or F1’). Hence, four types of comparisons were done, namely F1 female *vs S*. *haematobium* and *S*. *bovis* female, F1’ female *vs S*. *haematobium* and *S*. *bovis* female, F1 male *vs S*. *haematobium* and *S*. *bovis* male, and finally F1’ male *vs S*. *haematobium* and *S*. *bovis* male. Each of these comparisons allowed quantifying genes belonging to the five expression profiles as defined and described above.

### Transcriptome annotation and functional analysis

In order to annotate our newly assembled transcriptome, we used previous annotations from *S*. *haematobium* reference genome in combination with a *de novo* approach using different programs hosted either on the Galaxy instance of the IHPE laboratory or of the European server [62,63]. First, we extracted nucleotide sequences of each transcript we assembled previously using Gffread ([58], Galaxy Version 2.2.1.3+galaxy0). We then translated our transcripts sequences using TransDeCoder with default parameters ([64], Galaxy Version 5.5.0+galaxy2) in order to extract all Open Read Frames (ORF). Over the 66,012 assembled transcripts, 51,428 contained an ORF. Translated sequences were then annotated with Interproscan ([65], version 5.59–91.0+galaxy3) using default parameters. The annotation file in gff3 format was then converted to a tsv format using AGAT [66] in order to extract Gene Ontology (GO) terms, gene products terms and associate them to their corresponding gene. Note that a gene could be associated with several gene products if they coded for several transcripts and/or if they contained several ORFs. Finally, we also transferred to our assembly the annotations from the reference genome when genes were matching. Our annotation hence resulted in 10,256 annotated genes (with a product or a GO term) over the total of 16,840 genes assembled (61% overall annotation rate, 37% annotation rate based on GO terms only). The annotation rate obtained was a little lower than that of *S*. *haematobium’s* reference genome (48% for GO terms annotations [51]), which is likely due to novel assembled genes that were absent from annotation databases.

Regarding the functional analysis, we conducted classic exact fisher enrichment tests using the corresponding parameters in the Rank Based Gene Ontology Analysis suit of scripts [67] (https://github.com/z0on/GO_MWU; with default parameters, but absValue = 0.001, and input file coded in binary with “1” indicating that the gene belongs to the gene set and “0” that it does not) and run on the IHPE Galaxy instance. Exact fisher enrichment tests compared within each GO term the number of genes from a defined gene set to the number of genes from the reference to test whether this GO contains significantly more genes from the gene set compare to genes from the reference. P-values were further adjusted for multiple comparisons and significant GO terms were selected at a 5% FDR threshold. To ease results interpretation, we further selected non-redundant significant GO terms falling into biological processes and displayed Tree Map graphical representations using Revigo with list size set to small [68]. The functional analyses were conducted on gene sets of each hybrid expression profile categories (under- and over-expressed, intermediate, *S*. *haematobium*-like and *S*. *bovis*-like) independently for each hybrid cross (F1 and F1’) and each sex (female and male).

## Results

### Reference transcriptome assembly

A total of 16,840 genes were assembled in our new transcriptome. According to the Gffcompare report that compared our transcriptome assembly to *S*. *haematobium* gene models, only a few features (i.e., exon, intron, intron chain, transcript or genes) which were found in the reference annotation were not exactly matching those of our assembly (high Sensitivity value, S1 Table). On the contrary our assembly presented features that were not found in *S*. *haematobium* gene models (precision values were lower notably at the transcript level, S1 Table). No gene or transcript from the reference were missing from our assembly (S2 Table) and 14,668 transcripts of the 14,698 reference transcripts had a perfect match with one of our assembled transcript (S2 Table). A total of 5095 of gene in our assembly were totally novel (none of their transcripts matched with those of the reference, S2 Table). We hence conducted further analyses on the 16,840 genes we assembled since they contained features specific to our input RNA-seq data.

### Gene expression profiles

**Fig 2:**
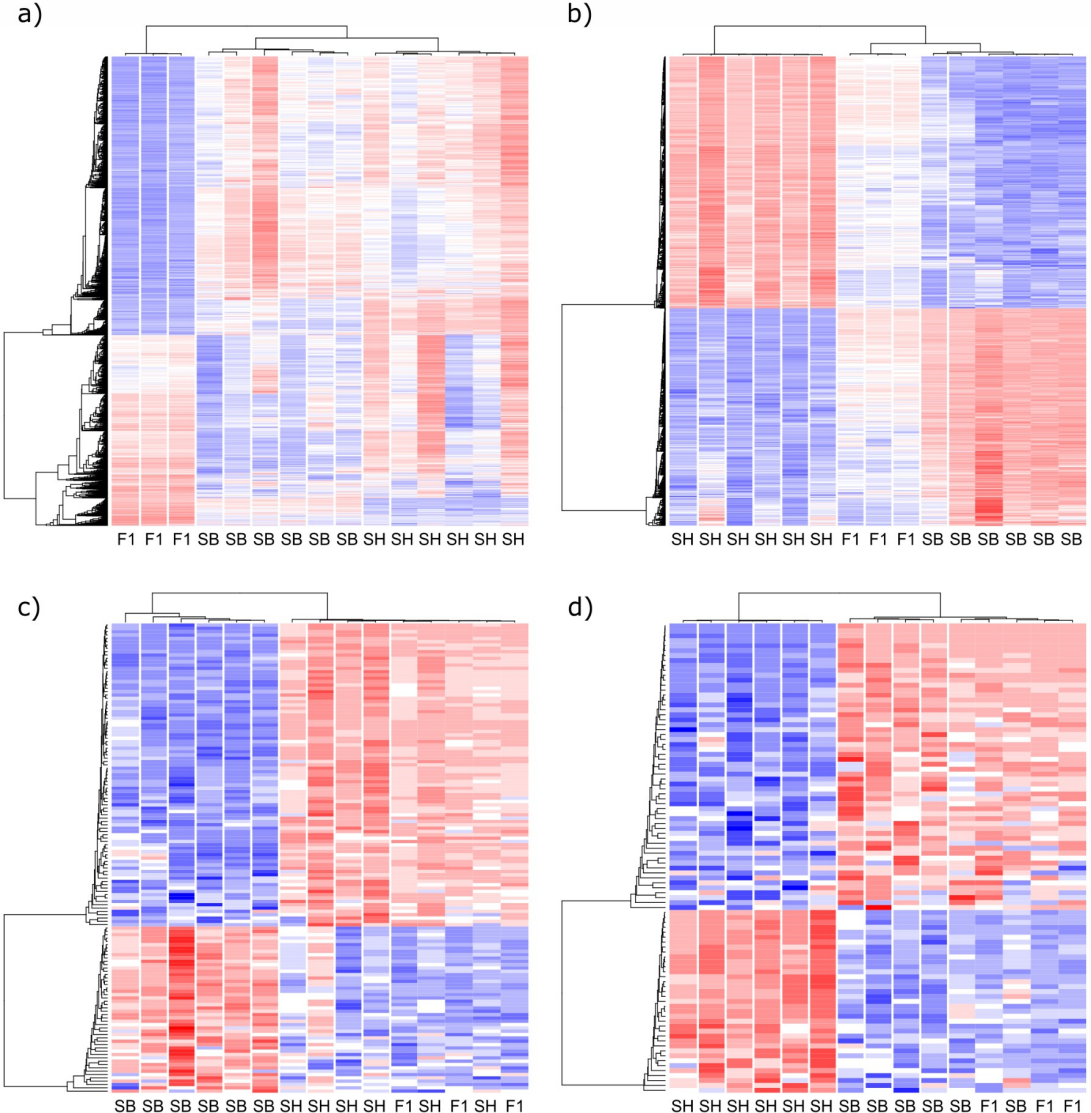
Heatmaps illustrating the gene clustering within each hybrid expression profiles and presenting as example F1 females (F1) compared to *S*. *haematobium* (SH) and *S*. *bovis* (SB) females: under and over-expressed profiles (a), intermediate profiles (b), *S*. *haematobium*-like profile (c) and *S*. *bovis*-like profile (d). See S3–S5 Figs for heatmaps of expression profiles of F1’ females, F1 males and F1’ males.

**Fig 3:**
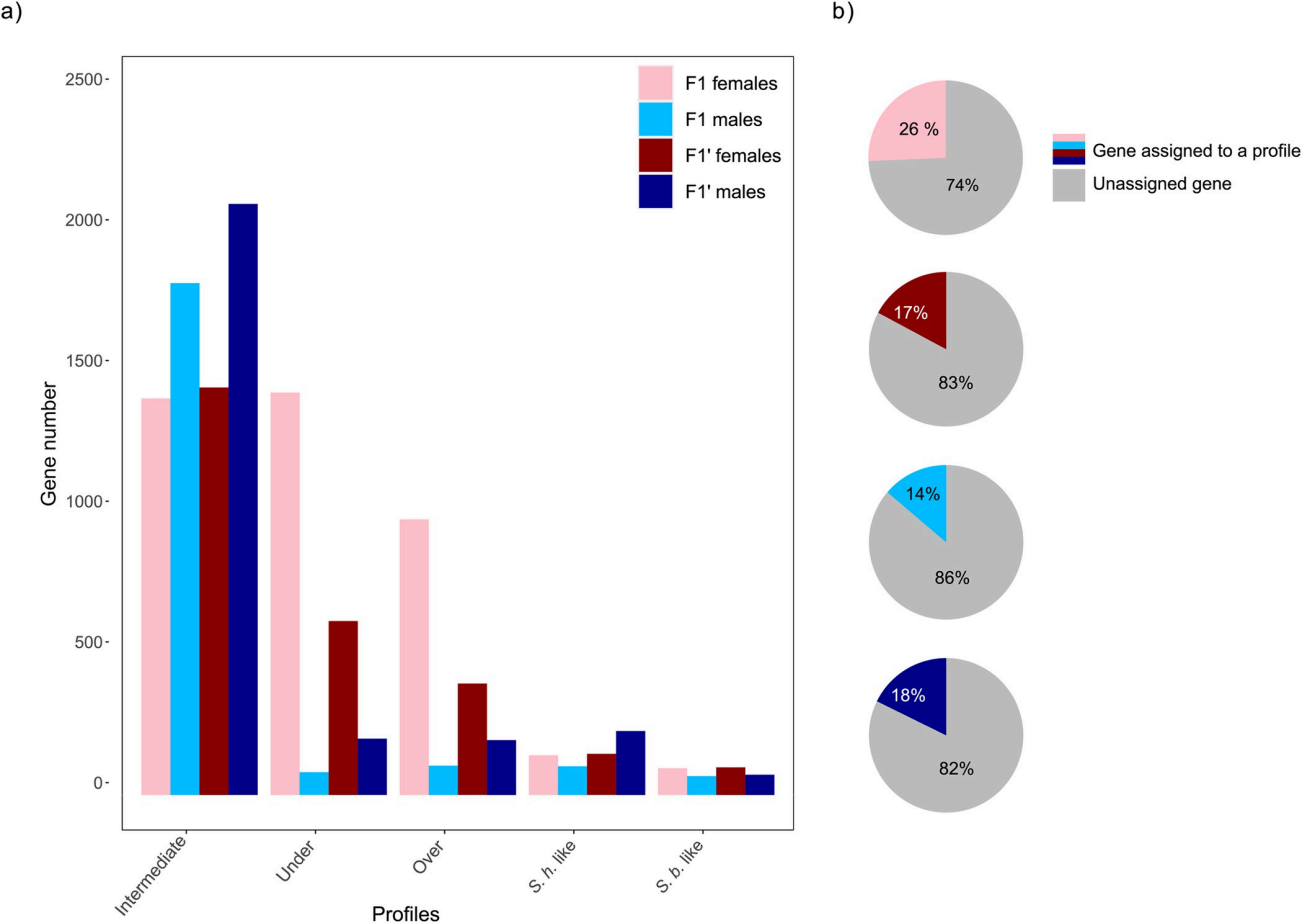
a) Barplot showing for males and females of each hybrid cross, the number of genes falling into the over-expressed, under-expressed, intermediate, *S*. *haematobium* (*S*.*h*.) and *S*. *bovis* (*S*.*b*.) like expression profiles. b) Pie charts showing the ratio between genes assigned to a gene expression profile and unassigned genes within each cross and sex.

The Principal Component Analysis conducted on parent and hybrid samples revealed that one F1 replicate clustered apart from others and in the wrong sex cluster hence suggesting that this sample was probably contaminated, we thus excluded it from further analysis (S2 Fig). Sex was the main segregating factor (first axis of a PCA conducted on all gene expression, S2 Fig), and, as expected, F1 samples were clustering between *S*. *haematobium* and *S*. *bovis* samples, (S2 Fig). We categorized five typical gene expression profiles being, *over-expressed*, *under-expressed*, *intermediate*, *S*. *haematobium-like* or *S*. *bovis-like* (see Figs 2 and S3–S5). After removing 1,070 lowly expressed genes (i.e., transcripts with less than 10 counts across all samples) from the initial 16,840 genes of the transcriptome, 15,770 genes were considered in the gene expression analysis. Overall, 7,100 genes (45%) were assigned to a hybrid expression profile (i.e., under-expressed, over-expressed, intermediate or like one or the other parental species) and 8, 670 (55%) genes were not assigned to a particular hybrid expression profile (those genes were either differentially expressed between the hybrid offspring and parental lines but without satisfying the criterions of each expression profile or were genes that were not differentially expressed at all, neither between hybrids and parental lines nor between *S*. *haematobium* and *S*. *bovis*, Sheet A in S1 File). The most abundant expression profile was the intermediate profile (i.e., 3,723 genes, 24% of investigated genes) whereas the less abundant profiles were the *S*. *haematobium-*like profile and the *S*. *bovis-*like profile with 592 (4%) and 319 (2%) genes respectively (Fig 3A, Sheet A in S1 File). Over- and under-expressed profiles included 1,501 (10%) and 1,912 (12%) genes respectively (Sheet A in S1 File). Interestingly for over- and under-expressed profiles, a substantial part of those genes was found in females and more frequently in F1 cross than in F1’ cross (see Fig 3A). Also, very few genes falling into each of the other expression profiles were common between crosses (i.e., between F1 and F1’) and between sexes (i.e., between males and females, see S6 Fig). We found common genes between crosses and sexes mostly for intermediate profiles (8.1% of the genes having an intermediate profile, see S6 Fig). Specifically, 9.1% of genes belonging to the intermediate profile were both found in F1 and F1’ females and 18.2% were both found in F1 and F1’males. Some over-expressed genes were found both in F1 and F1’ females (7.3% of all over-expressed genes) and some under-expressed genes were found both in F1 and F1’ females (17.2% of all under-expressed genes). On average 81% of genes were unassigned to a particular gene expression profile within each cross and sex (Fig 3B).

### Functional analysis

**Fig 4:**
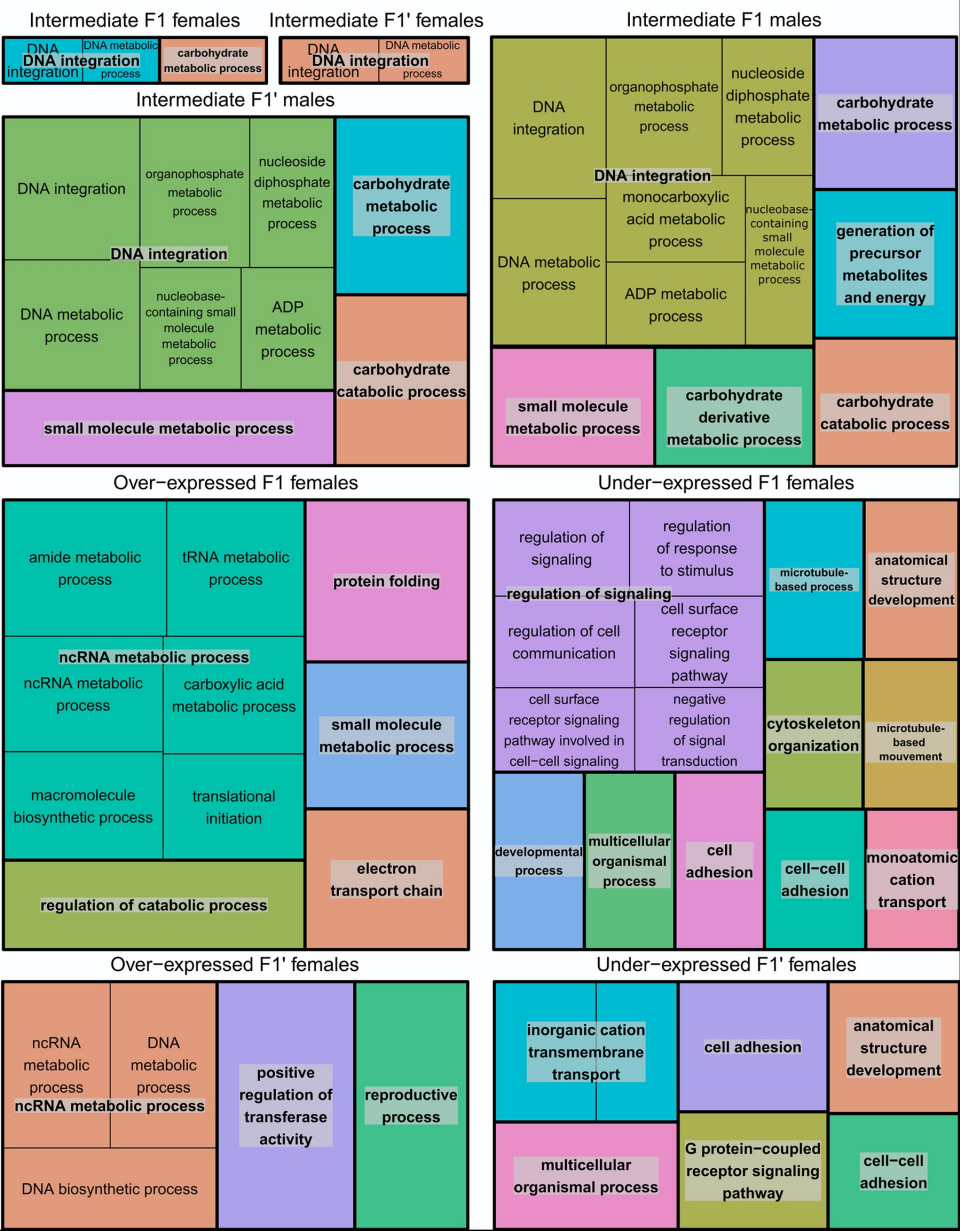
Tree maps of non-redundant biological processes enriched in genes having an intermediate profile, in genes having an over-expressed expression profile and in genes having an under-expressed expression profile. Relevant sex and cross are indicated above each tree map.

Gene Ontology (GO) terms were found enriched in genes belonging to eight sets of gene expression profiles compared to the overall pool of gene considered (16,840 total genes). Precisely, GO terms were significantly enriched with genes belonging to intermediate expression profiles (F1 and F1’ females, F1 and F1’ males), over-expressed profiles (both F1 and F1’ females) and under-expressed profiles (both F1 and F1’ females, see Sheets B and C in S1 File and Fig 4). Conversely, no GO terms were found enriched with gene sets corresponding to *S*. *haematobium* and *S*. *bovis* like expression profiles or with gene sets corresponding to male over- and under-expressed profiles.

GO terms enriched in genes belonging to intermediate gene expression profiles were quite congruent between cross and sexes and including core terms such as *DNA integration* and *Carbohydrate metabolic process* (Fig 4). GO terms enriched in over-expressed genes were also congruent between F1 and F1’crosses both including the core term *non-coding RNA metabolic process*. Interestingly the GO term *Reproductive process* was found enriched in over-expressed genes in F1’ females but not in F1 females. Finally, GO terms enriched in under-expressed genes were once again shared between F1 and F1’ females, both including terms such as *anatomical structure development*, *cell-adhesion*, *cell-cell adhesion*, and *multicellular organismal process*.

It is worth noting, that when directly examining the annotated gene product belonging to intermediate expression profiles, a high number of genes associated with retrotransposon activity were found (See Sheet B in S1 File). Specifically, in the entire transcriptome 773 genes were tagged with the term *reverse transcriptase* as product. Up to 278 (36%) of these were genes belonging to intermediate expression profile, while 84 (11%), 50 (6%), 20 (3%) and 17 (2%) were found in over-expressed genes, under-expressed genes, *S. bovis*-like expressed genes and *S. haematobium*-like expressed genes respectively. Hence the GO term *DNA integration* in intermediate expression profiles is most likely linked to retro-transposon activity.

## Discussion

In this study, we aimed at describing first generation *S*. *haematobium x S*. *bovis* hybrids compared to their parents at the transcriptomic level. We were able to assemble up to 16,840 genes and assign their expression profiles into five categories namely: intermediate, over- and under-expressed, *S*. *haematobium*-like and *S*. *bovis*-like. The intermediate expression profile was the most common, but both under- and over-expressed profiles appeared relatively abundant in F1 females (*S*. *haematobium* mother and *S*. *bovis* father) and to a lesser extent in F1’ females (*S*. *haematobium* father and *S*. *bovis* mother). While genes falling into each expression profile were cross- and sex-specific, the corresponding biological processes were congruent between crosses and sexes. We specifically showed an over-representation of *DNA integration* function in intermediate profiles that could be reflecting an enhanced retro-transposon activity as expected in hybrids. In addition, biological processes associated with heterosis such as *Reproductive process* and more generally *Development* were represented among over- and under-expressed gene expression profiles. We hence provide new insights on the molecular machinery expressed within first generation hybrids while providing some explanation for the progeny viability and potential heterosis arising from *S*. *haematobium x S*. *bovis* hybridization.

Over the 15,770 genes tested we found that up to 7,100 genes were differentially expressed in *Schistosoma* hybrids compared to at least one of the parental species suggesting that some changes in gene expression patterns occurred in *S*. *haematobium* x *S*. *bovis* first generation hybrids. First generation hybrids benefit from an entire new set of alleles brought from the combination of the two parental species. This can lead to a “genomic shock” as proposed by Barbara McClintock [69] which usually results in a disruption of gene expression regulation and in the activation of genetic mobile elements, leading to new patterns of gene expression for part of genes in hybrids [70], as observed here in this study. We further show that the most prevalent expression profile when considering all sexes and crosses was the intermediate expression profile also referred to as additive [42,43]. These genes with an intermediate expression profile are associated with processes related to *DNA integration* and more specifically to retro-transposon activity (36% of retro-transposase were falling into intermediate expression profile), suggesting an expression of transposable elements in first generation hybrids that is different from the ones in the parental lines [71]. These transposable elements could be linked to the “genomic shock” expected in first generation hybrids [69]. However, this would need further investigation, starting with a complete annotation of these elements, to better decipher the control mechanisms that have been disrupted, as well as the impact that their expression might have in shaping the hybrid genomic landscape in subsequent generations.

The fact that among hybrid expression profiles the intermediate one was the most frequent also denotes that compatibility between *S*. *haematobium* and *S*. *bovis* is probably largely maintained. Intermediate expression of a gene in the hybrid offspring would be expected when the expression level of each parental allele is maintained (without interference due to different species origin) so their average expression at the gene level is indeed at an intermediate level. This would only be possible if the transcriptomic machineries, such as transcription factors and trans-regulatory factors, in *S*. *haematobium* and *S*. *bovis*, remain compatible one to the other. *Schistosoma haematobium* and *S*. *bovis* are generally known to present genomes that are highly permeable one to the other [24]. Recent studies tend to demonstrate various levels of introgression (up to 23% in the case of *S*. *haematobium x S*. *bovis* [23,25,41]) in many schistosome species [23,26,27,72,73]. At the transcriptomic level, mating between *S*. *haematobium* and *S*. *bovis* have also been found to trigger only few differential gene expressions which also suggests that the two species remain strongly co-adapted in terms of gene regulation [24]. In our case similarly, genes assigned to a particular expression profile were very specific to the hybrid sex and to the cross direction. Within a specific cross and sex, the actual number of genes that were not assigned to a profile actually represented the majority of genes (on average 81%). These unassigned genes could be either differentially expressed genes but not typical of any expression profiles or they could be genes that do not show any differential expression between either the two parental lines or the hybrids and parental lines. Thought, the investigation of such conserved genes in terms of expression was not the focus of the current study, we estimated that they corresponded to 20% to 26% (depending on the cross and the sex) of the total 15770 genes analysed. This showed that a substantial portion of the transcriptome was not significantly impacted by hybridization. We thus suggest that the abundant number of genes unassigned to a particular hybrid expression profile along with the intermediate gene expression profiles being more prevalent among *S*. *haematobium* and *S*. *bovis* hybrids might be another facet of the signature of the high permeability that still exists between *S*. *haematobium* and *S*. *bovis* genomes.

*Schistosoma* first generation hybrids are not only fertile, but some offspring may show heterosis (higher infectivity, production of more numerous and larger eggs, [24,27,28,40]). Heterosis is commonly expected to result either from complementary expression of two inbred parental species (dominance model) or from synergetic interaction between parental alleles (over-dominance model) [9]. In terms of gene expression, each model is expected to translate either into over-expressed and under-expressed profiles or into parent-like expression profile respectively (i.e., overall non-intermediate expression profiles, [4,5,43,74,75] but see [42] for intermediate expression profiles associated with heterosis). In our study, we show that over- and under-expressed profiles are more prevalent than parent-like expression profiles and thus contain more genes that are likely to be associated to heterosis in the case of *Schistosoma* hybrids. Consequently, if heterosis is confirmed in *S*. *haematobium* and *S*. *bovis* first generation hybrids, we suggest that at the molecular level it would be better explained by an over-dominance model in which synergetic expression of parental alleles occurs. Furthermore, over- and under-expression profiles were more abundant in females than males (see Fig 2). In this study we were thus able to detect gene expression profiles compliant with heterosis expression in females, however, the lack of direct gene candidates for heterosis in male does not necessary mean that heterosis in *S*. *haematobium x S*. *bovis* male hybrids does not occur, including for instance at a different stage of parasite’s the life cycle.

We further provide in this study some insights about the biological processes that are impacted in first generation hybrids compared to parental lines. Genes differentially expressed in hybrids were different depending on the sex and the cross, but they were overall involved in congruent biological processes across crosses and sexes within a particular hybrid expression profile. We already mentioned that genes having an intermediate expression profile were associated with *DNA integration processes* and potentially transposable elements activity which might relate to the genomic shock that hybrids undergo through an activation of mobile elements [69]. Some other biological processes from genes expressed differentially in hybrids could on the other hand be related to heterosis. Heterosis is indeed a complex multigenic trait for which no consensus has yet been found across the tree of life. Some authors suggest that it may be due to a cumulative effect of the differential expression of a variety of genes involved in one or several metabolic pathways affecting yield, energy use efficiency and/or reproductive success [76,77]. We found that *Carbohydrate metabolic process* was associated with genes expressed at an intermediate level in hybrids compared to parental lines. Carbohydrate metabolism could indeed relate to the parasite’s ability to manage their food intake and/or regulation and use of energy. Similarly, we found that some biological processes that could be related to heterosis were enriched with the genes susceptible to lead to exacerbated expression of such processes in hybrids compared to parental lines (i.e., over-expressed genes but also under-expressed genes). First, among the experimental F1’ females we found *Reproductive process* being over-expressed which could directly mirror observations made at the phenotypic level as in other *Schistosoma* species [78], although higher yields of eggs in *S*. *haematobium* x *S*. *bovis* first generation hybrids needs to be confirmed (but see [20]). Furthermore, *non-coding RNA metabolic process* was also enriched in both F1 and F1’ females. Non-coding RNA have been suggested to greatly impact regulatory networks and were also directly associated with heterosis in rice hybrids [79]. Otherwise, regarding biological processes enriched with under-expressed genes, those were mostly related to signaling pathways (eg. *Regulation of signaling* and *G-protein-coupled receptor signaling pathway*, *cell adhesion* and *Anatomical structure development*, Fig 4). While those processes are more difficult to interpret, one could speculate that these processes could relate to the mating process between the male and female. In particular, females are known to undergo strong developmental changes upon mating and this process relies on critical molecular cross talk between the two partners [80,81]. Under-expressing processes related to mating might contribute to ease future sex interactions within hybrids or with parental lines. The biological processes enriched in genes differentially expressed in first generation hybrids between *S*. *haematobium* and *S*. *bovis* can hence be linked to the genomic shock expected after hybridization but noticeably also to heterosis and compatibility of two co-adapted species.

It is worth noting that for a long time hybrid vigor has been studied independently from hybrid incompatibility. As such, studies aiming at identifying the molecular basis of heterosis are still often restricted to model species or species of agroeconomic interest [4–6,75]. However, some studies focusing on the molecular basis of hybrid incompatibilities have investigated wild species [82,83]. Interestingly both type of studies highlighted the presence of genes having non-additive profiles but gave them a very contrasting interpretation (basis of heterosis on one hand and proof of mis-regulation in the other hand (eg., [6,83]). These observations along with our results seem to support the idea that heterosis is one facet of hybrid incompatibility. Here our findings can be interpreted at the light of the idea exposed in [7] stating that hybrid vigor can result from a mis-regulation of multiple process, potentially involved in trade-offs with reproduction and growth functions. Our results suggest a differential activation of transposable elements in hybrids compared to parental lines and those mobile elements are known to reshuffle gene regulatory networks [71]. On the other hand, we also identified congruent biological processes that can be directly associated with heterosis and that can reduce functional incompatibilities. We suspect DNA integration processes and transposable elements to be a consequence of hybridization that could contribute to establish new patterns of gene expression. The outcome of those new patterns of gene expression in hybrids can generally be on a continuum between heterosis and hybrid incompatibility but precisely understanding their adaptive effects in a particular environment should be the focus of further studies.

In conclusion, in this study we relied on a transcriptomic analysis of experimental first-generation hybrids between two *Schistosoma* species to unravel the molecular machinery of hybridization and the potential basis of the spectrum of hybrid incompatibility and heterosis. Our results are congruent with a maintained compatibility between the parental lines and also the probable occurrence of heterosis in first generation *S*. *haematobium* x *S*. *bovis* hybrids, potentially mediated through changes in gene regulation networks. We indeed found that 7,100 genes in hybrids were expressed differently than at least one of the parental species hence suggesting that changes in gene regulatory networks occur after hybridization. The predominance of hybrid genes expressed at intermediate levels compared to the parental lines highlights that compatibility between the parental species is still largely maintained. We also report that in *S*. *haematobium x S*. *bovis* first generation hybrids, gene expression patterns following an over-dominant model (hence compatible with heterosis through synergetic expression of each parental allele) included genes coding for functions related to reproductive success. Our study is hence meaningful in the context of recent discoveries around introgression events between *Schistosoma* species, while providing insights about what could occur in a scenario of ongoing hybridization.

## Supporting information

S1 Fig Principal Component Analysis based on 11 microsatellite markers (Sh9, Sh3, C102, Sh1, Sh14, C111, Sh13, Sh11, Sh2, Sh5 and Sh12). The blue dots are *S*. *haematobium* parasites (miracidia) recovered from human in Cameroun [37]. The grey dots are adult *S*. *bovis* parasites collected from cattle [45]. The orange dots are the *S*. *haematobium* parasites (cercariae) that formed the strain at the origin of the crosses. First axis (68%)–Second axis (32%).
(TIF)

S2 Fig Principal Component Analysis of transcriptiomic data from *S*. *haematobium*, *S*. *bovis* or their first-generation reciprocal hybrids (F1: female *S*. *haematobium* x male *S*. *bovis;* F1: female *S*. *bovis* x male *S*. *haematobium*). We can see that samples cluster by sex (PC1) and parasite species origin (PC2). One male F1 sample clustered apart from others and has been excluded from further analysis.
(TIF)

S3 Fig Heatmaps illustrating the gene clustering within each hybrid expression profiles in F1’ females (F1’) compared to *S. haematobium* (SH) and *S. bovis* (SB) females: under and over-expressed profiles (a), intermediate profiles (b), *S. haematobium*-like profile (c) and *S. bovis-like* profile (d).
(TIF)

S4 Fig Heatmaps illustrating the gene clustering within each hybrid expression profiles in F1 males (F1) compared to *S. haematobium* (SH) and *S. bovis* (SB) males: under and over-expressed profiles (a), intermediate profiles (b), *S. haematobium*-like profile (c) and *S. bovis*-like profile (d).
(TIF)

S5 Fig Heatmaps illustrating the gene clustering within each hybrid expression profiles in F1’ males (F1’) compared to *S. haematobium* (SH) and *S. bovis* (SB) males: under and over-expressed profiles (a), intermediate profiles (b), *S. haematobium*-like profile (c) and *S. bovis*-like profile (d).
(TIF)

S6 Fig Venn plots showing shared genes involved in each hybrid expression profiles between the two hybrid crosses (F1 and F1’) and the two sexes. (TIF)

S1 Table Statistics extracted from gffcompare report when comparing our assembly to the reference genome of *S*. *haematobium*. (XLSX)

S2 Table Details from the gffcompare output on the correspondence between transcripts and genes from our assembly and of the *S*. *haematobium*‘s reference genome. At the transcript level are presented the number of transcripts that were found in the reference but not in our assembly (missing), the number of transcripts that exactly matched the full intron chain of transcripts of the refence (exact match), the number of transcripts that matched imperfectly (eg, not the full intron chain) transcripts of the reference (non-exact match), the number of transcripts that mapped unproperly to transcripts of the reference (unreliable match, i.e. including possible pre-mRNA fragment, match on the opposite strand likely resulting from a mapping error and no-actual overlap) and that did not match at all any transcripts of the reference (novel). The same statistics are provided at the gene level. We also present the total number of transcripts and gene in our assembly and in the reference genome of *S*. *haematobium*.
(XLSX)

S1 File **Sheet A**. Table summarising for each gene assembled from *S. haematobium*, *S. bovis* and their first-generation hybrids RNA-seq, their expression profile (if any), the cross and sex in which their expression profile has been measured, relevant Log2 Fold Changes (LFC) for contrasts between hybrids and parents along with their associated False Discovery Rate (FDR). In bold are figured the LFC and FDR used when establishing a profile for a given gene. **Sheet B**. Table summarising for each gene assembled from *S. haematobium*, *S. bovis* and their first-generation hybrids RNA-seq, their expression profile (if any), the cross and sex in which their expression profile has been measured, GO terms annotated, putative gene product and (when any) the associated gene name and ID from the reference genome of *S. haematobium*. **Sheet C**. Table summarising the result of the gene ontology analysis and giving for each enriched Gene Ontology their associated expression profile, the level of the GO term, the total number of sequences having this GO term, the name of the GO term and their corrected p-value.
(XLSX)
