# Supplementary figures and images for: Schistosoma haematobium and Schistosoma bovis first generation hybrids undergo gene expressions changes consistent with species compatibility and heterosis
Source: PLoS Negl Trop Dis. 2024 Jul 2;18(7):e0012267. doi: 10.1371/journal.pntd.0012267 (PMC11249247; doi:10.1371/journal.pntd.0012267)

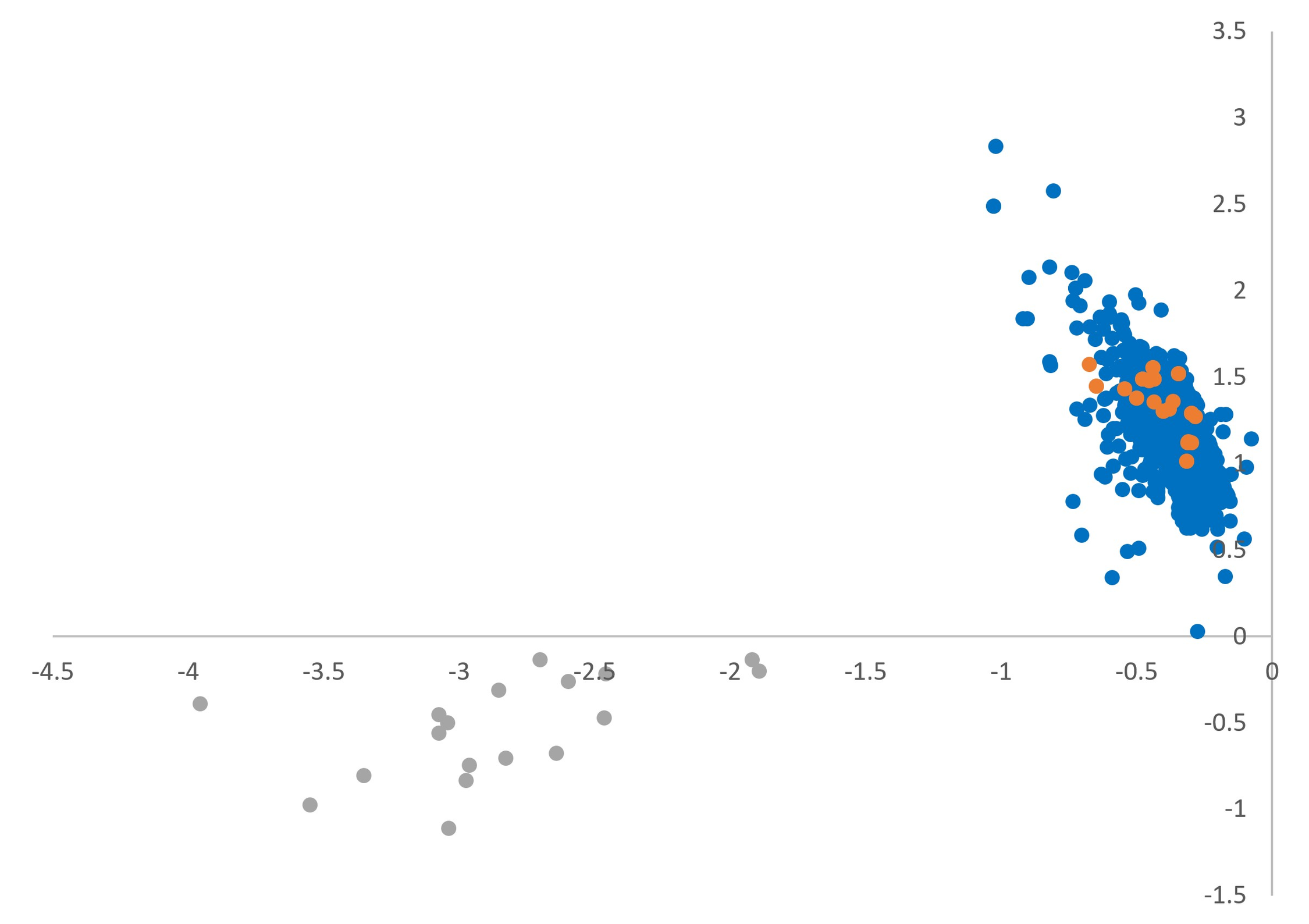

Supplement: S1 Fig — The blue dots are S. haematobium parasites (miracidia) recovered from human in Cameroun [37]. The grey dots are adult S. bovis parasites collected from cattle [45]. The orange dots are the S. haematobium parasites (cercariae) that formed the strain at the origin of the crosses. First axis (68%)–Second axis (32%). (TIF) [file pntd.0012267.s001.tif]

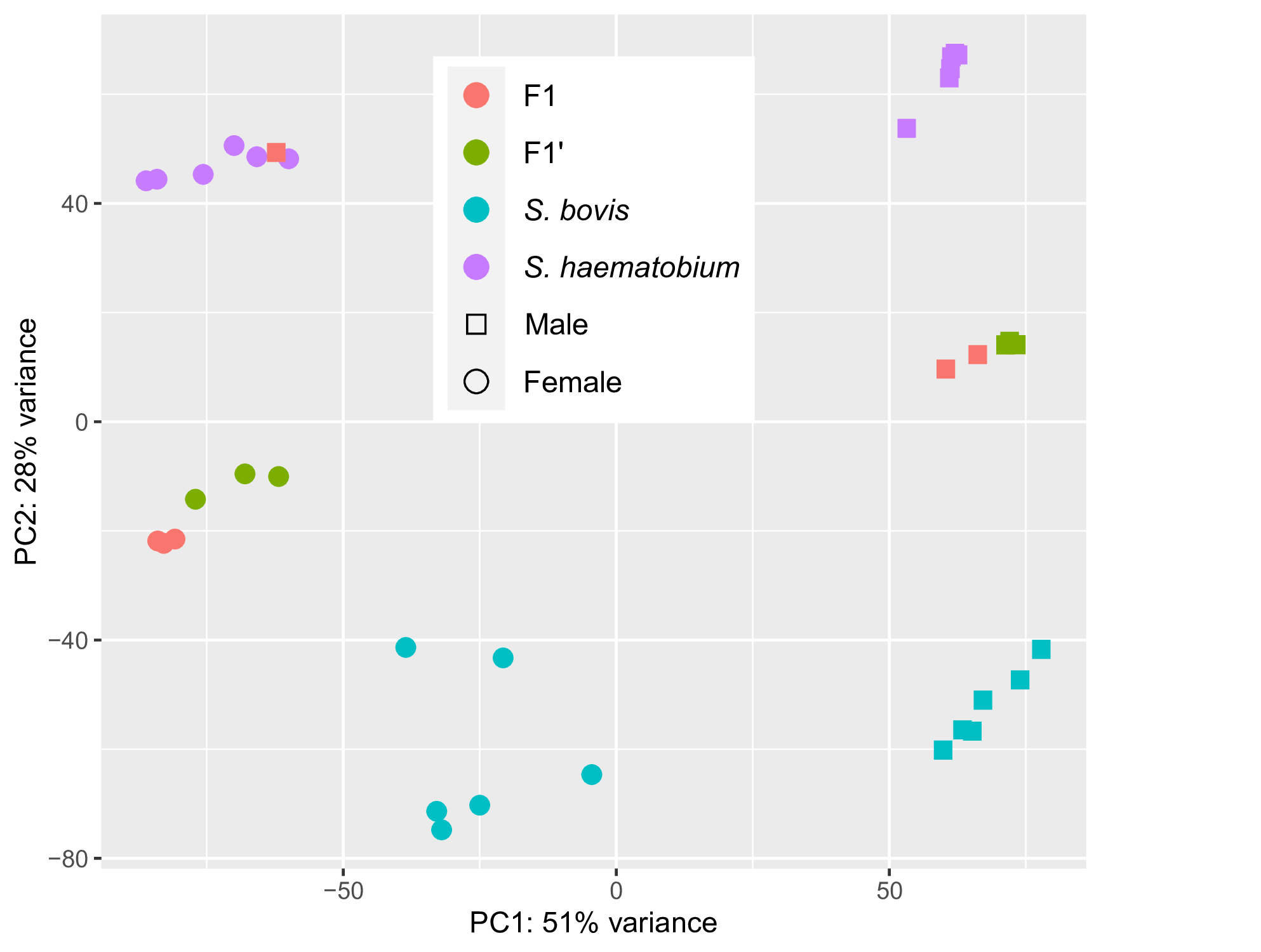

Supplement: S2 Fig — We can see that samples cluster by sex (PC1) and parasite species origin (PC2). One male F1 sample clustered apart from others and has been excluded from further analysis. (TIF) [file pntd.0012267.s002.tif]

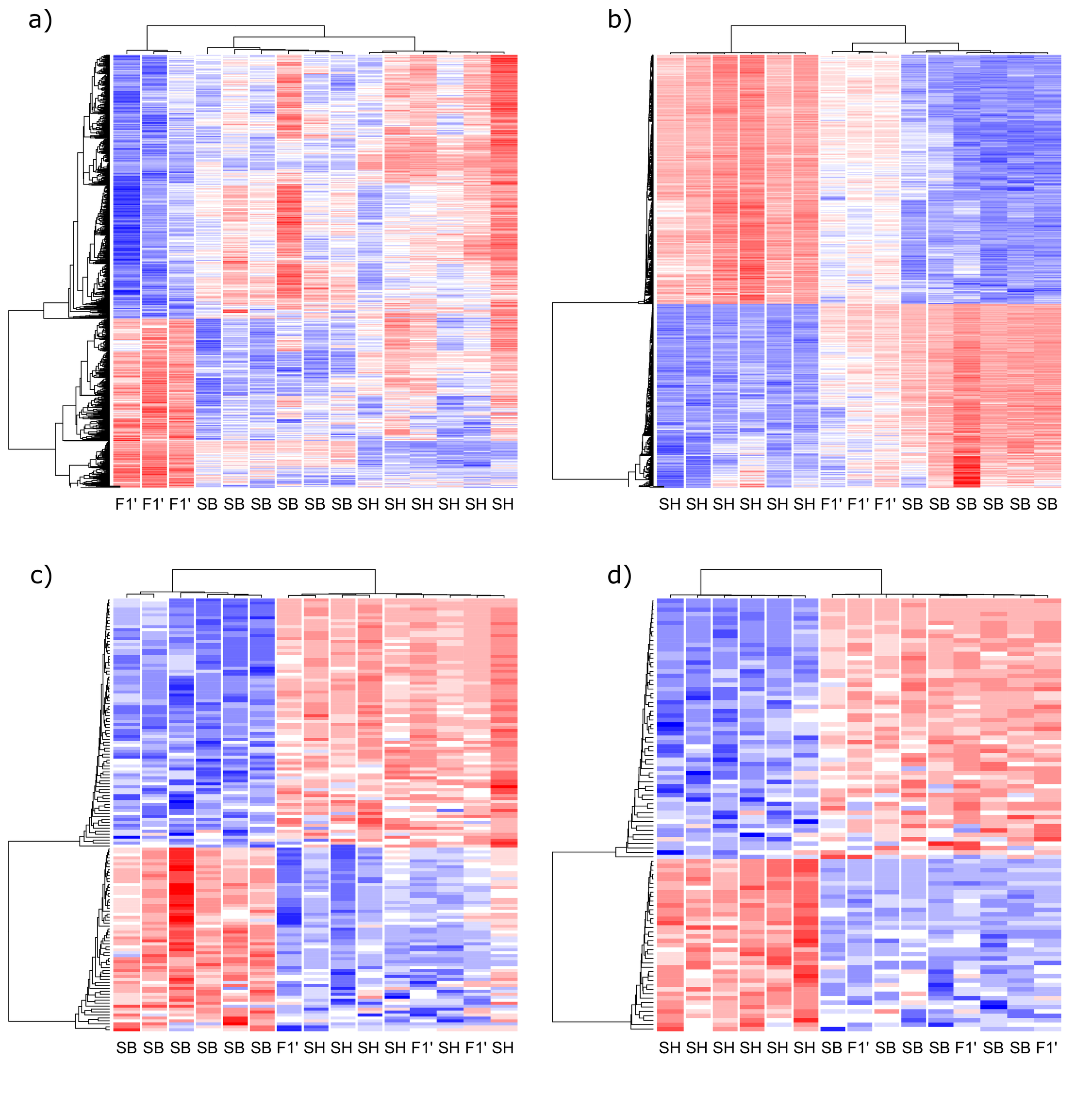

Supplement: S3 Fig — Heatmaps illustrating the gene clustering within each hybrid expression profiles in F1’ females (F1’) compared to S. haematobium (SH) and S. bovis (SB) females: under and over-expressed profiles (a), intermediate profiles (b), S. haematobium-like profile (c) and S. bovis-like profile (d). (TIF) [file pntd.0012267.s003.tif]

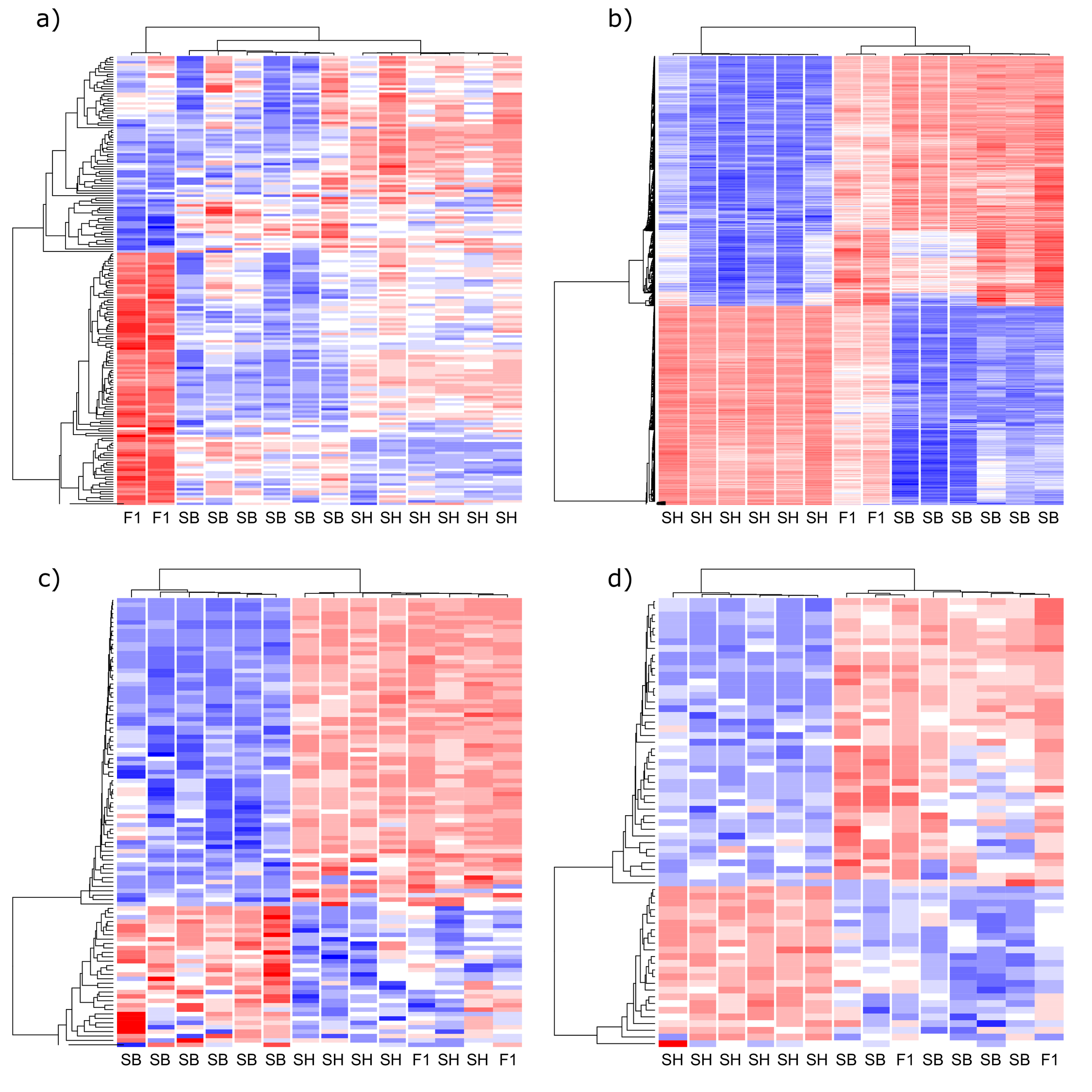

Supplement: S4 Fig — Heatmaps illustrating the gene clustering within each hybrid expression profiles in F1 males (F1) compared to S. haematobium (SH) and S. bovis (SB) males: under and over-expressed profiles (a), intermediate profiles (b), S. haematobium-like profile (c) and S. bovis-like profile (d). (TIF) [file pntd.0012267.s004.tif]

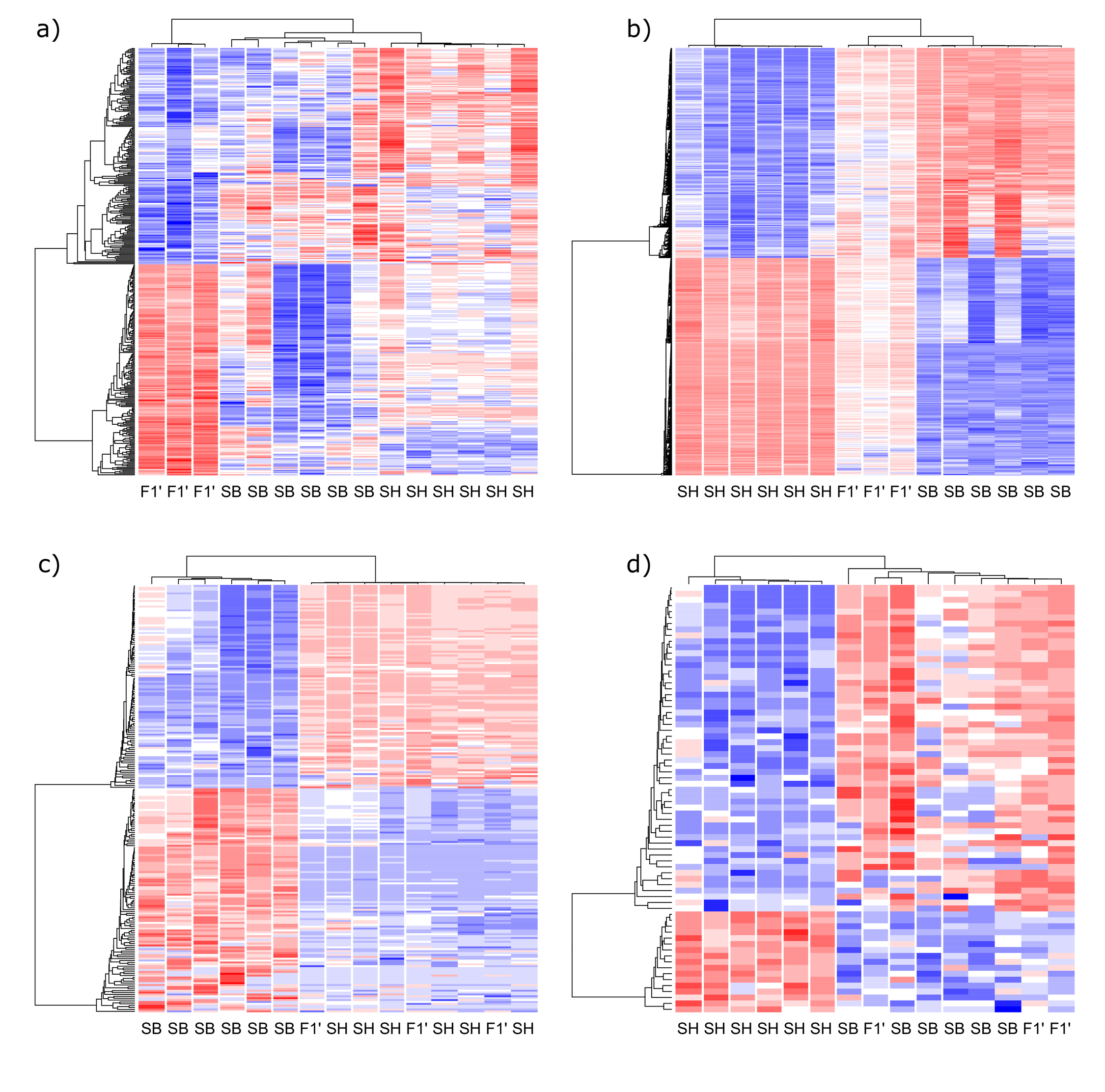

Supplement: S5 Fig — Heatmaps illustrating the gene clustering within each hybrid expression profiles in F1’ males (F1’) compared to S. haematobium (SH) and S. bovis (SB) males: under and over-expressed profiles (a), intermediate profiles (b), S. haematobium-like profile (c) and S. bovis-like profile (d). (TIF) [file pntd.0012267.s005.tif]

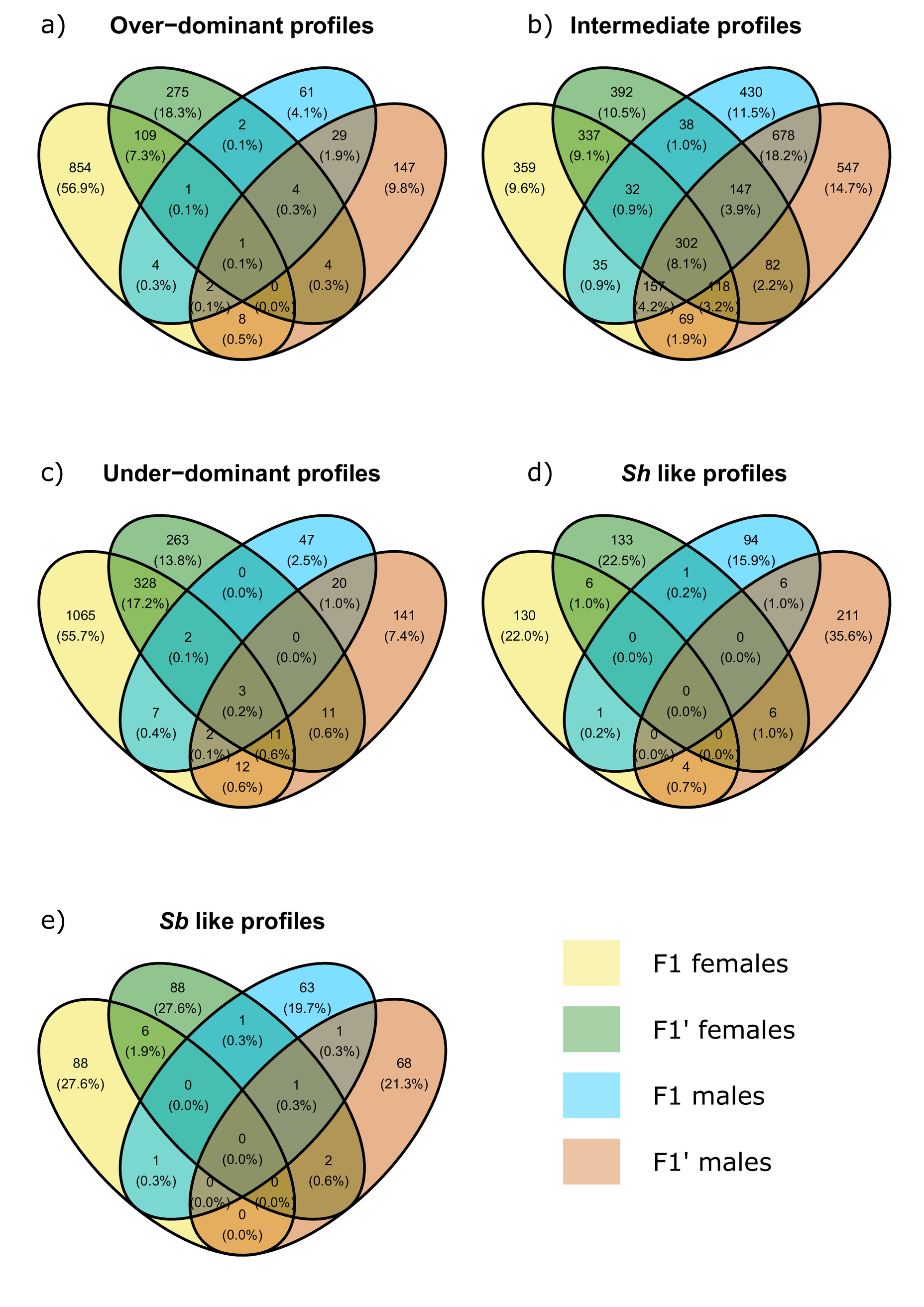

Supplement: S6 Fig — (TIF) [file pntd.0012267.s006.tif]
